# Supplementary material for: Body Size Awareness and Modular Self-Representation in Reedfish (Erpetoichthys calabaricus): Near-Field Passability Judgments
Source: Animals (Basel). 2025 Nov 7;15(22):3231. doi: 10.3390/ani15223231 (PMC12649500; doi:10.3390/ani15223231)
Supplement: Supplementary file 1 [file animals-15-03231-s001.zip › Supplementary materials-11.5/Exp-2-first approaches/interaction_plot.pdf]

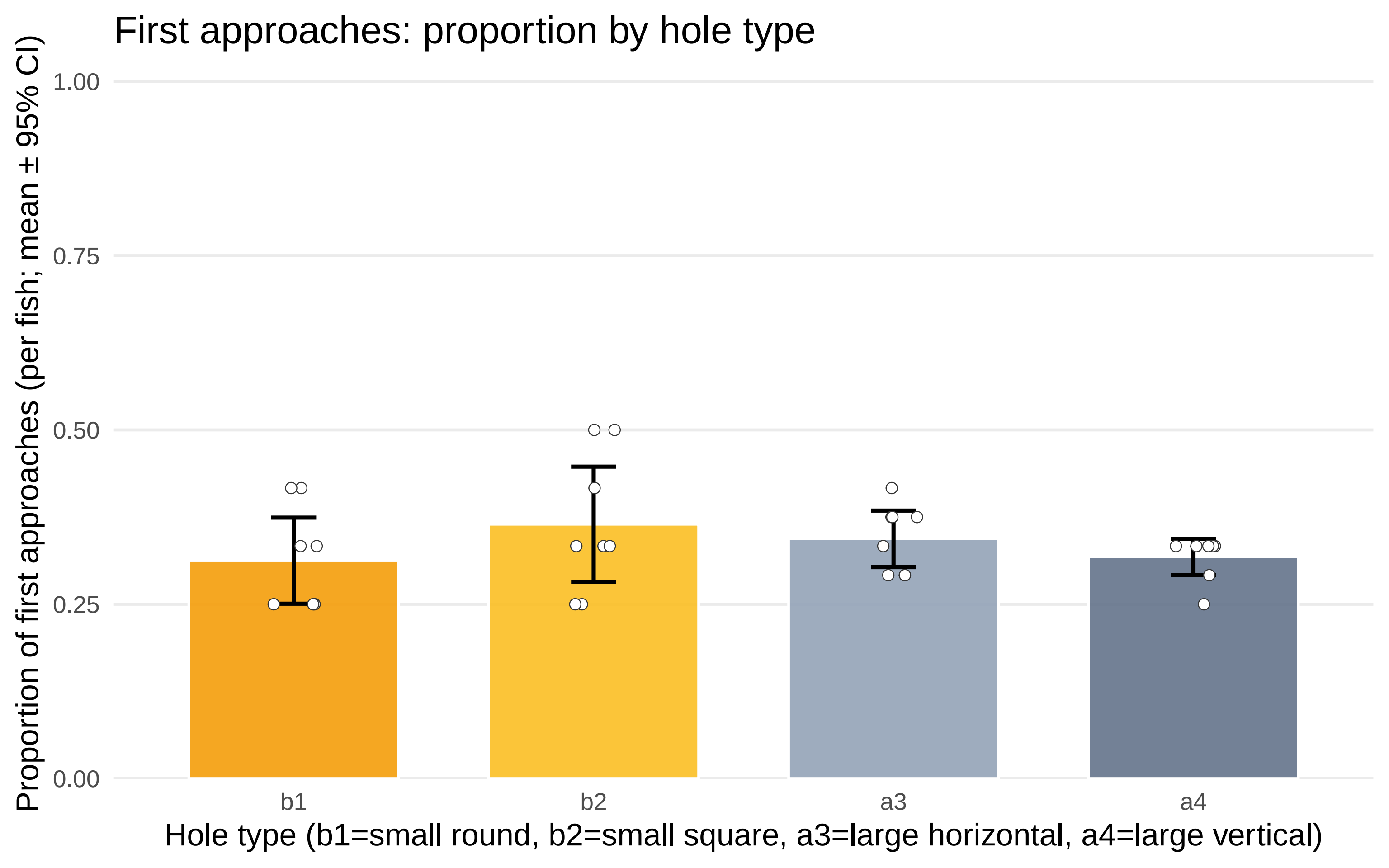
First approaches: proportion by hole type
Proportion of first approaches (per fish; mean ± 95% CI)
1.00
0.75
0.50
0.25
0.00
b1
b2
a3
a4
Hole type (b1=small round, b2=small square, a3=large horizontal, a4=large vertical)
