# Supplementary figures and images for: Body Size Awareness and Modular Self-Representation in Reedfish (Erpetoichthys calabaricus): Near-Field Passability Judgments
Source: Animals (Basel). 2025 Nov 7;15(22):3231. doi: 10.3390/ani15223231 (PMC12649500; doi:10.3390/ani15223231)

Fish choices by hole size and position

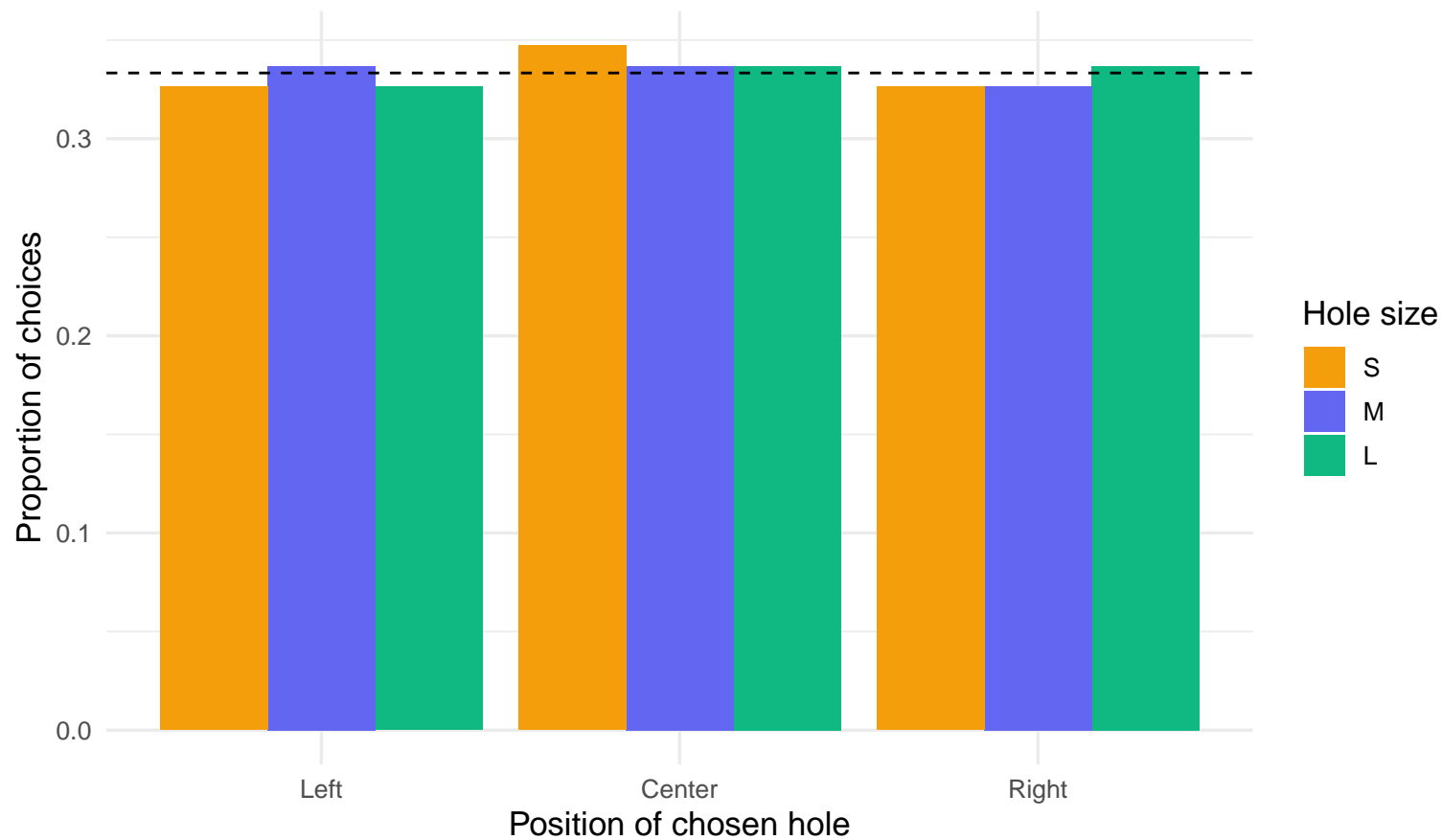

Supplement: Supplementary file 1 [file animals-15-03231-s001.zip › Supplementary materials-11.5/Exp-1/interaction_plot.pdf]

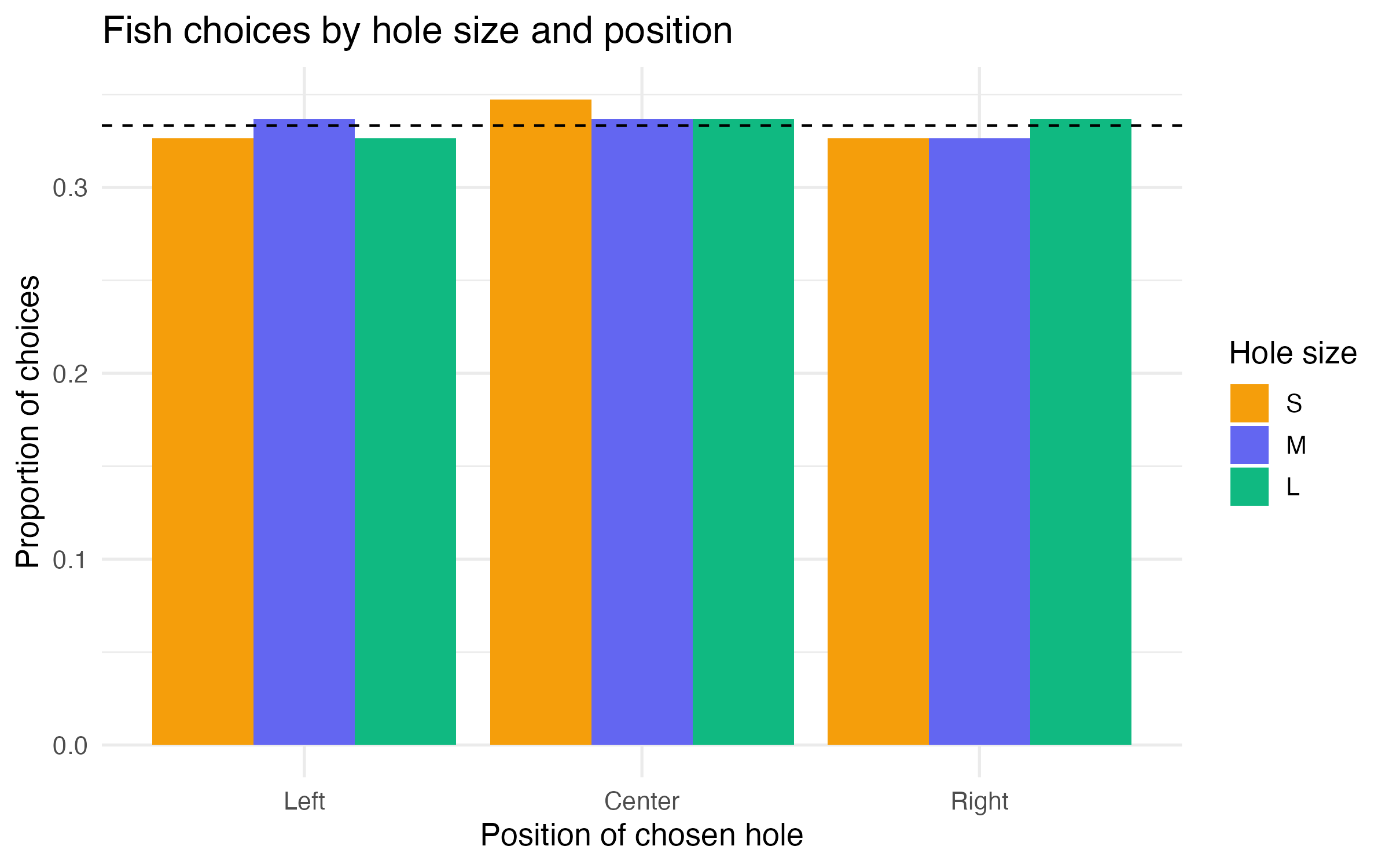

Supplement: Supplementary file 1 [file animals-15-03231-s001.zip › Supplementary materials-11.5/Exp-1/interaction_plot.png]

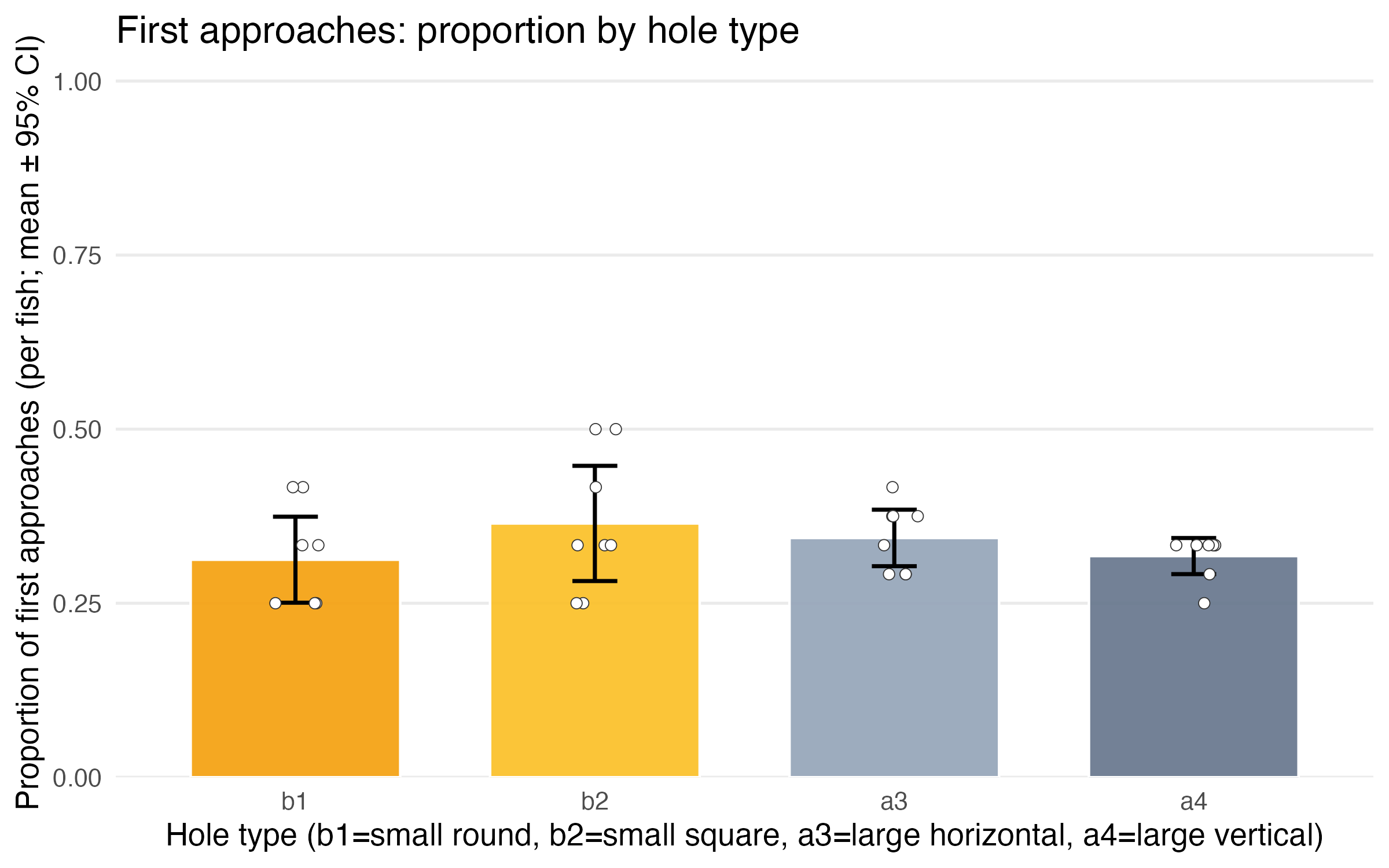

Supplement: Supplementary file 1 [file animals-15-03231-s001.zip › Supplementary materials-11.5/Exp-2-first approaches/interaction_plot.png]

Proportion of choices by hole type

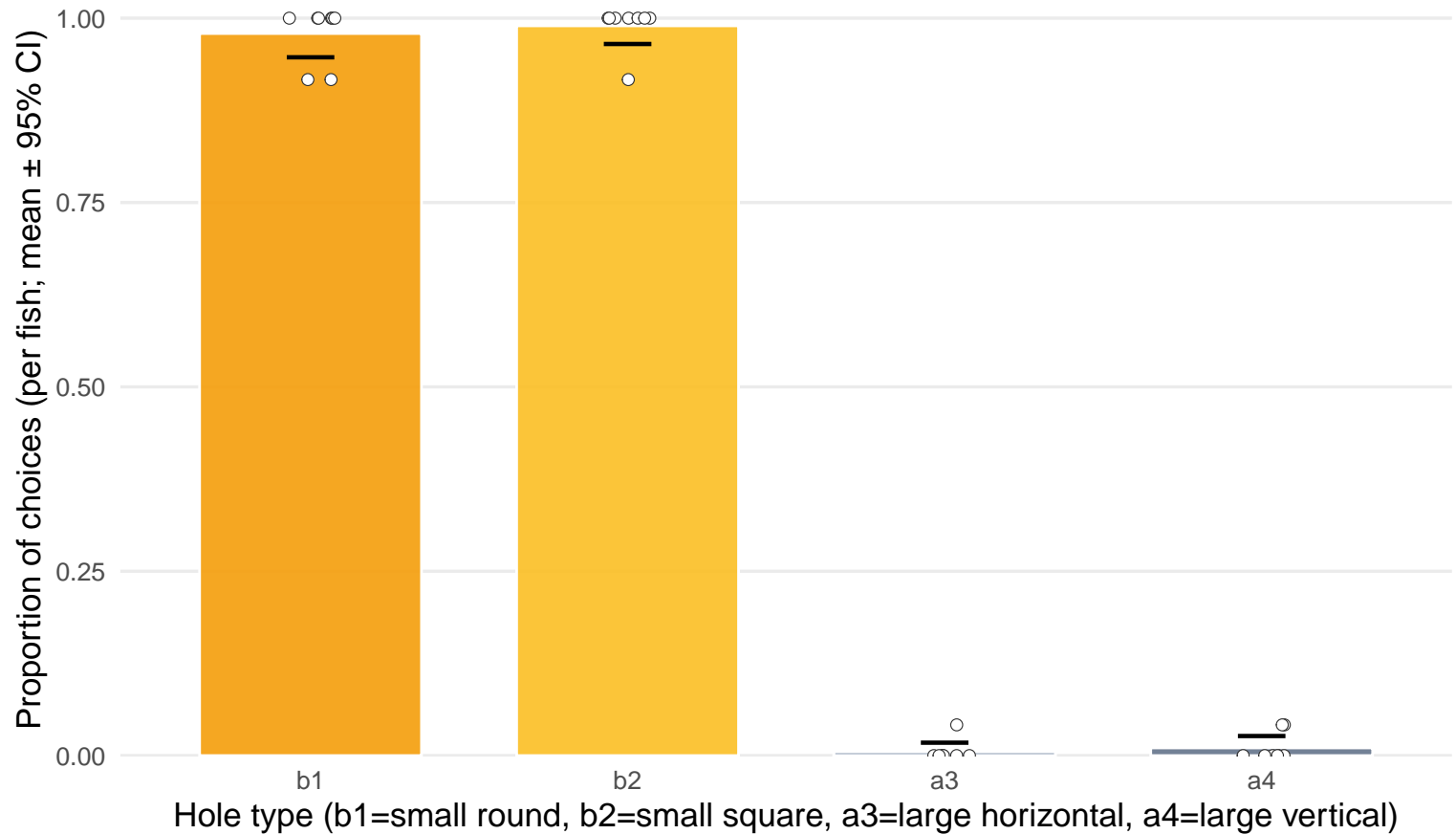

Supplement: Supplementary file 1 [file animals-15-03231-s001.zip › Supplementary materials-11.5/Exp-2-first attempts to pass/interaction_plot.pdf]

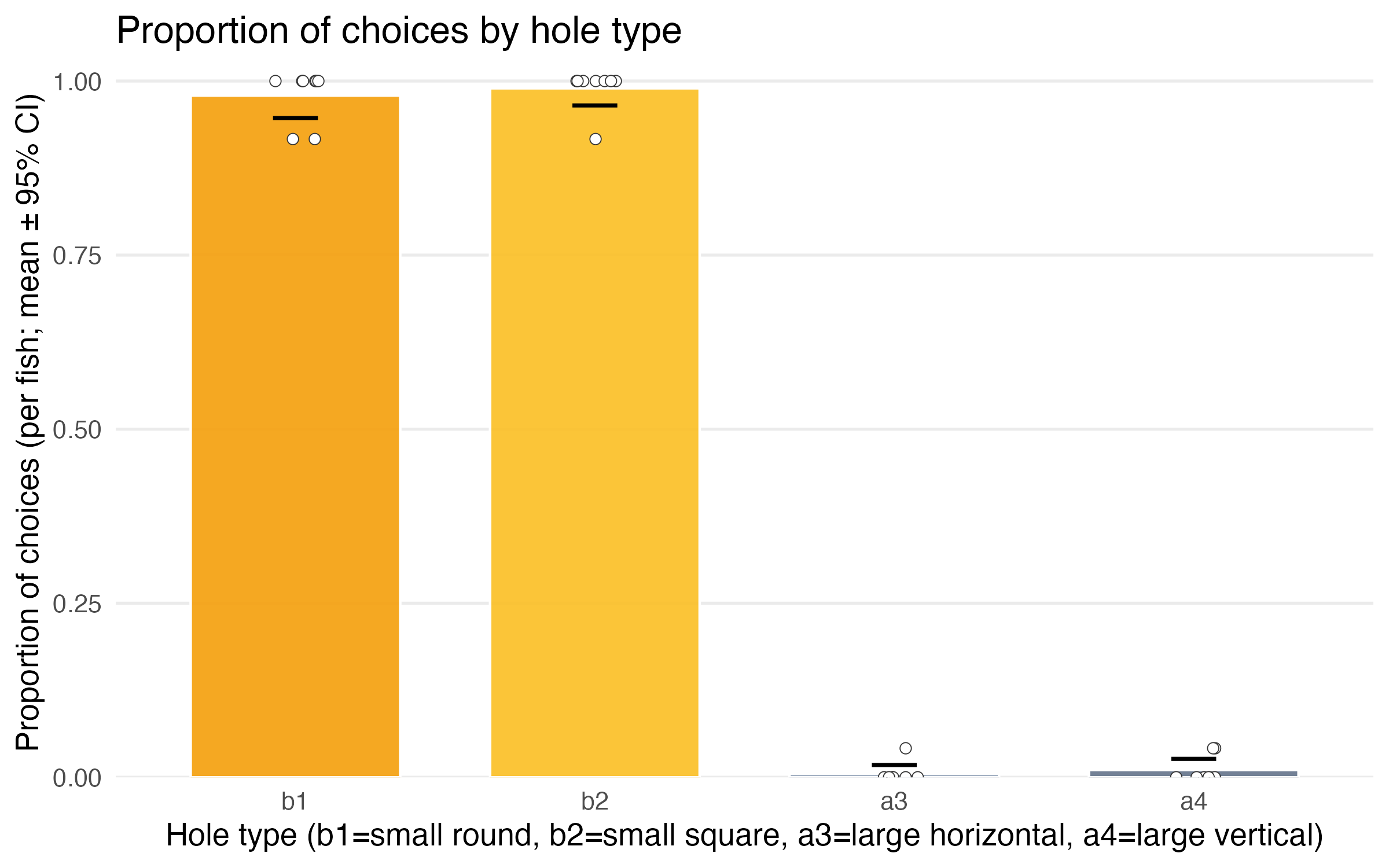

Supplement: Supplementary file 1 [file animals-15-03231-s001.zip › Supplementary materials-11.5/Exp-2-first attempts to pass/interaction_plot.png]
